# Li Jump Diffusion and Long-Range Transport in Garnet Single Crystals: Spanning the kHz–GHz Range

**DOI:** 10.1021/acs.chemmater.6c00979

**Published:** 2026-07-16

**Authors:** Jana Königsreiter, Jonas Spychala, Gergö Horvath, Kunimitsu Kataoka, Florian Stainer, Junji Akimoto, H. Martin R. Wilkening

**Affiliations:** † 27253 Graz University of Technology, Institute of Chemistry and Technology of Materials (NAWI Graz), Stremayrgasse 9, 8010 Graz, Austria; ‡ National Institute of Advanced Industrial Science and Technology (AIST), 1-1-1 Higashi, Tsukuba, Ibaraki 305-8565, Japan; § 52747 National Institute for Materials Science (NIMS), 1-1 Namiki, Tsukuba, Ibaraki 305-0044, Japan

## Abstract

Understanding and controlling Li-ion transport in garnet-type oxides is central to advancing solid electrolytes for all-solid-state energy storage systems. Garnet electrolytes have long been recognized for their high ionic conductivity, and the availability of large single crystals now enables a direct, frequency-resolved view of Li-ion dynamics over a broad time window. In such structurally and chemically homogeneous systems, responses from electrical and nuclear magnetic resonance measurements are expected to align, yielding a consistent picture of frequency-dependent Li-ion hopping processes. Here, we investigate single-crystalline Li_6.5_La_3_Zr_1.5_Ta_0.5_O_12_ and probe ion dynamics from the kHz to GHz range using a combination of ^7^Li nuclear spin relaxation (NSR) and electrical conductivity spectroscopy. Long-range transport (5 × 10^–4^ S cm^–1^ at 293 K) is consistently described by activation energies in the range of 0.41 to 0.47 eV, whereas localized ion dynamics, observed, e.g., by laboratory-frame NSR, are associated with much lower barriers of approximately 0.22 eV. In comparison with single-crystalline Li_6_La_3_ZrTaO_12_, which exhibits reduced ionic mobility, we propose that changes in ionic mobility are compensated by a higher effective charge-carrier concentration *N*_c_ in systems with lower Li contents. For Li_6.5_La_3_Zr_1.5_Ta_0.5_O_12_, the Li^+^ mobility is higher by approximately 1 order of magnitude; however, the resulting conductivity only slightly exceeds that of Li_6_La_3_ZrTaO_12_, presumably due to a lower density of mobile charge carriers. This finding highlights that *N*_c_ is a key parameter to consider in these systems that indeed sensitively governs the practical ionic conductivity.

## Graphical abstract

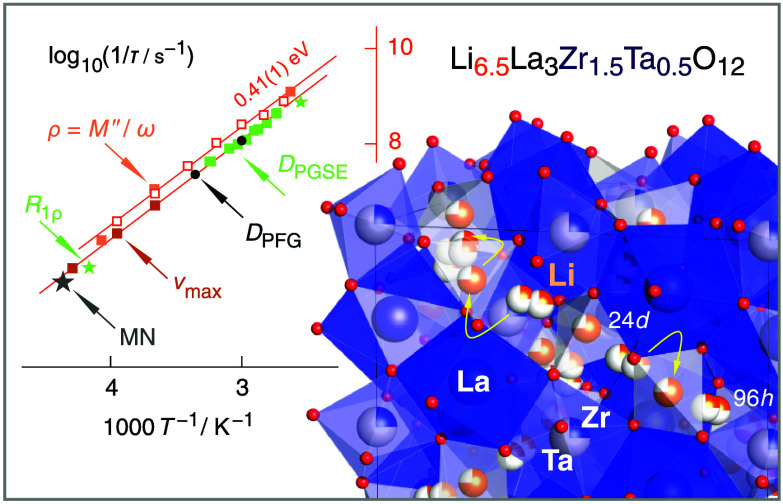

## Introduction

Electrochemical energy storage underpins the transition to a low-carbon energy system, from electric mobility to the integration of intermittent renewables.^1,2^ Rechargeable lithium batteries remain the leading technology owing to their high energy density and mature manufacturing base.^3,4^ Yet, further gains in safety, energy density, and lifetime are increasingly constrained by the liquid electrolytes used in commercially available cells, which introduce flammability risks, limit operating windows, and complicate cell design at high voltages and with lithium metal anodes.^5–7^ Solid-state batteries promise to overcome several of these limitations by replacing the flammable liquid with an inorganic or polymer solid electrolyte, thereby improving intrinsic safety, enabling thin lithium metal anodes, and potentially raising both volumetric and gravimetric energy densities.^8–11^ Realizing these benefits requires solid electrolytes that combine high room-temperature Li-ion conductivity, wide electrochemical stability windows, chemical and mechanical compatibility with electrodes, and robust processability.^8,12–15^

Among the classes of inorganic solid electrolytes,^8,16–23^ oxide garnets have emerged as prime candidates due to their wide electrochemical stability against high-voltage cathodes, resistance to moisture compared to sulfides, and competitive ionic conductivities.^24–28^ The Li-garnet family, exemplified by Li_7_La_3_Zr_2_O_12_ (LLZO)^29^ and its aliovalently substituted derivatives,^30^ hosts a three-dimensional Li substructure within a rigid oxide framework that supports fast Li-ion migration at and above ambient temperature.^29,31^ Chemical substitutions at the Zr site (e.g., Ta, Nb; see Figure 1) and controlled Li stoichiometry tune both the carrier concentration and the topology of accessible migration pathways, allowing optimization of bulk transport.^32–34^ Despite these advances, the microscopic nature of Li transport in garnets remains complex, sometimes exhibiting dynamic anomalies even in macroscopic diffusion measurements.^35^ We propose that the partial occupancy of Li sites creates site-to-site energy variations, giving rise to an energy landscape with frequency-dependent ion dynamics.

**Figure 1:**
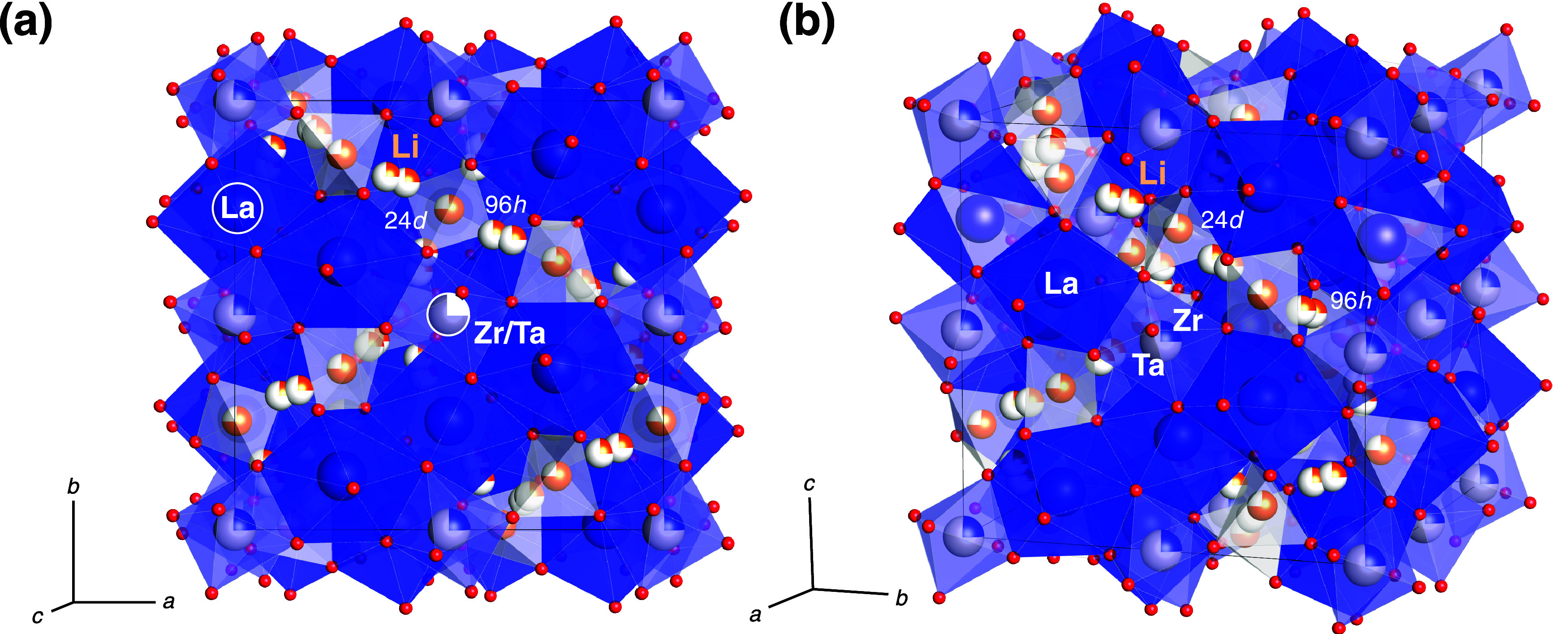
Crystal structure of cubic Li_6.5_La_3_Zr_1.5_Ta_0.5_O_12_ (*Ia*3*d*) shown in two views, with the La and Zr/Ta sublattices highlighted. Li ions occupy the 24*d* and split 96*h* sites, which together form a three-dimensional diffusion network for Li^+^. For the average structure of the sample, Kataoka and Akimoto^30^ report a split site even for the Li ions occupying the 24*d* void, which would further increase the degree of Li^+^ occupation disorder in Li_6.5_La_3_Zr_1.5_Ta_0.5_O_12_ and shorten the average Li–Li distance.

A critical barrier to disentangling these effects is methodological. Impedance spectroscopy^36^ is the workhorse technique for assessing ionic conductivity and activation energies associated with long-range transport.^37–39^ However, despite its ability to probe local ion dynamics, it is less commonly used to investigate short-range ion motion. In polycrystalline garnets the interpretation is often confounded by grain boundaries, secondary phases, and even interfacial polarization, which can mask or distort the intrinsic bulk response and degrade the precision of extracted parameters.^40–42^ While careful microstructural control and equivalent-circuit modeling mitigate these issues, unambiguous access to the bulk is greatly facilitated in single crystals,^43,44^ where grain boundaries are absent and electrode polarization can be separated over broad frequency–temperature windows.^45^ Furthermore, previous studies on Al-containing LLZO have shown that chemical inhomogeneities and local compositional variations, which are more likely to occur in powder samples than in single crystals, can measurably affect Li-ion dynamics and the corresponding NMR response.^43^

In contrast, nuclear magnetic resonance (NMR) is inherently bulk-sensitive when microcrystalline rather than nanocrystalline samples are considered. Nanocrystalline materials, however, contain a much larger fraction of interfacial regions, whose spin density may be high enough to allow their ion dynamics to be detected. The method directly probes the motion of, e.g., ^7^Li spins (spin-quantum number *I* = 3/2) via fluctuations of magnetic dipolar and electric quadrupolar interactions. Without requiring specific sample geometries or postprocessing, NMR can interrogate single crystals, ceramics, glasses, and polymers alike, thereby providing a unified view across materials classes. Diffusion-induced nuclear spin relaxation (NSR) and line-shape narrowing report on motional time scales from kilohertz to gigahertz and on distinct motional correlation functions tied to local jump processes.

If conducted in the so-called direct-current time window, conductivity spectroscopy, by comparison, reflects the long-range, percolative aspects of motion encoded in charge transport and correlates with tracer diffusion through quantities such as the Haven ratio.^46^ A system that allows both techniques, *viz* NMR and conductivity spectroscopy, to be applied under well-defined, grain-boundary-free conditions therefore offers a unique opportunity to compare local and long-range descriptors of ion dynamics on equal footing.

Single-crystalline Li_6.5_La_3_Zr_1.5_Ta_0.5_O_12_ (LLZTO) provides such a platform.^47,48^ In contrast to Al-stabilized LLZO, where Al directly occupies Li sites and can lead to a broader distribution of local Li environments, Ta substitutes on the Zr sublattice and therefore affects Li-ion transport more indirectly through charge compensation and structural modifications.^43^ In this composition, the Li content is adjusted through a tailored Zr:Ta ratio rather than by introducing Al^3+^ on the Li sublattice as in the case of Al-bearing LLZO.^34,49^ Avoiding Al maintains the intrinsic topology and, thus, reduces extrinsic perturbations to the migration network^43^ while still enabling a high number density *N*_c_ of ionic charge carriers. Given the crystallographic complexity of LLZTO with Li ions occupying the 3D network of interconnected 24*d* and 96*h* sites,^30^ we nevertheless expect an irregular potential energy landscape in which Li ions encounter several local barriers. This view is expected to manifest as frequency-dependent dynamic signatures and as distinct activation energies associated with localized versus long-range motion. Probing these processes as a function of temperature is essential to map the “crossover” from local site-to-site hopping to extended diffusion and to quantify the effective energy barriers governing each regime.

By avoiding the various Al-related perturbations, Li_6.5_La_3_Zr_1.5_Ta_0.5_O_12_ offers the opportunity to investigate how subtle changes in Li content and vacancy concentration influence ion dynamics within the garnet framework. In comparison with Li_6_La_3_ZrTaO_12_,^48^ the higher Li concentration is expected to modify both the effective charge-carrier concentration and the topology of the Li diffusion network. Comparing these two closely related compositions therefore provides an opportunity to disentangle the roles of carrier concentration, activation energy, and microscopic ion-transport mechanisms in determining the overall ionic conductivity. Such insights are important for establishing composition-structure-dynamics relationships in garnet-type solid electrolytes. In this context, large, compositionally homogeneous single crystals provide an ideal platform for probing these effects, enabling a direct, frequency-resolved assessment of Li jump diffusion and transport across the kHz to GHz window while cleanly separating intrinsic bulk behavior from extrinsic microstructural effects.

By combining ^7^Li NMR relaxation with broadband conductivity spectroscopy on the same crystals, we can juxtapose observables that arise from different correlation functions, reconcile microscopic and macroscopic transport descriptors, and test for internal consistency across techniques. Comparative work on Al-containing LLZO single crystals has provided valuable insights into the role of dopants,^33,43,50^ and, as mentioned above, an earlier study on single crystalline Li_6_La_3_ZrTaO_12_, with lower Li content than that of the crystal studied here, explored related questions.^48^

The present work leverages this combination of material design and multimodal spectroscopy to build a coherent picture of Li-ion dynamics in LLZTO over a broad range of frequencies and temperatures. By aligning the bulk-sensitive NMR perspective on local jump processes with the impedance-derived view of long-range transport in single crystals, we aim to clarify how the energy landscape of garnets governs both short- and long-range mobility and to establish benchmarks for interpreting transport in more complex, polycrystalline systems.

## Experiment

Single-crystal rods of the composition Li_6.5_La_3_Zr_1.5_Ta_0.5_O_12_ were grown by floating-zone melting. Details of the synthesis procedure and structural features determined by single-crystal X-ray diffraction are reported elsewhere.^30^

^7^Li NMR spectra and NSR rates were recorded at a Larmor frequency of ω_0_/2π = 116 MHz using an Avance III Bruker digital NMR spectrometer connected to a shimmed 7 T wide-bore superconducting magnet (Bruker). All NMR measurements were carried out on intact single-crystal specimens; the crystals were not crushed into powder prior to the experiments. A Bruker high-temperature NMR probe, capable of operation up to 573 K, was employed. The sample temperature, up to 513 K in the present case, was regulated by a stream of dry, freshly evaporated dinitrogen gas and controlled via a Eurotherm temperature unit integrated into the spectrometer. Further details of the experimental setup and measurement techniques are reported elsewhere.^51–54^ Single pulse 90° experiments were used to acquire the ^7^Li NMR spectra at different temperatures; number of scans ranged from 4 to 64. Spin–lattice relaxation rates in the laboratory frame (*R*_1_≡1/*T*_1_) and in the rotating frame of reference (*R*_1ρ_≡1/*T*_1ρ_) were measured using the saturation-recovery and spin-lock techniques, respectively.^52,55^ The pulse sequences were: (i) 10 × (π/2) – *t*_d_ – (π/2) – acquisition, for *R*_1_; and (ii) (π/2) – p_lock_ – acquisition, for *R*_1ρ_.^52^ The length of the π/2 radio frequency pulse varied with temperature and ranged from 2.9 to 4.1 μs; the number of scans ranged from 4 to 32.

In sequence (i), an initial pulse train consisting of ten π/2 pulses separated by 80 μs was used to eliminate longitudinal magnetization (*M*_*z*_ = 0 at *t*_d_ = 0). The recovery of *M*_*z*_ toward equilibrium was then monitored as a function of the delay time *t*_d_. For *R*_1ρ_ measurements (ii), immediately after the initial π/2 pulse the transverse magnetization *M*_(*xy*)ρ_ oriented in the (*xy*)_ρ_ plane of the rotating coordinate system, was spin-locked by a radiofrequency field *B*_1_ = ω_1_/γ, where γ is the magnetogyric ratio. Note that *B*_1_ is much smaller than the static magnetic field *B*_0_ defining ω_0_. The decay of *M*_ρ_ during the locking period was monitored by recording the integrated area of the free induction decay as a function of the locking-pulse duration *t*_lock_. The spin-lock field strength was adjusted to ω_1_/2π = 20(1) kHz. This spin-lock frequency was chosen as a compromise between efficient spin locking and acceptable rf power dissipation during the long locking periods required for the relaxation measurements. Lower locking fields are more sensitive to offset effects and rf-field inhomogeneities, whereas substantially higher fields would increase the average rf power load and potential probe-heating effects. The recycle delay for *R*_1ρ_ experiments was set to at least 5 × *T*_1_. Both *R*_1_ and *R*_1ρ_ were extracted by fitting the magnetization transients *M*_*z*_(t_d_) and *M*_ρ_(*t*_lock_), respectively, using stretched-exponential functions: *M*_*z*_(*t*_d_) = *M*_0_ [1 – exp­[− (*t*_d_/*T*_1_)^φ^]] and *M*_ρ_(*t*_lock_) = *M*_0ρ_ exp­[− (*t*_lock_/*T*_1ρ_)^κ^]. The two stretching factors κ and φ were constrained to the interval [0, 1]. Where necessary, we employed a sum of two stretched exponentials (e.g., containing κ_1_, κ_2_ and the amplitudes *M*_ρ01_ and *M*_ρ02_) to account for deviations from a single stretched-exponential time behavior in spin-lock relaxation NMR.

To remove the surface layer of Li_2_CO_3_ formed during single-crystal cutting, the sample was dry-polished under an inert Ar atmosphere until the surface visibly cleared. The crystal was then heat-treated at 300 °C under dynamic vacuum to eliminate residual surface degradation. A second polishing step was performed after the heat treatment. Because the NMR experiments were performed on intact single crystals, possible surface-related contributions are expected to be negligible owing to the very small fraction of nuclei residing in near-surface regions. For the AC conductivity measurements we applied Au electrodes on a flat piece of the single crystal with cleaned surfaces. We used a Novocontrol Concept 80 broadband impedance spectrometer equipped with an α impedance analyzer and combined with an active ZGS cell interface (Novocontrol) to cover frequencies from 0.01 Hz to 10 MHz.^37^ The impedance measurements were performed under potentiostatic conditions using an rms excitation amplitude of 100 mV. Preliminary tests confirmed that this perturbation amplitude remained within the linear response regime of the investigated single crystals. For measurements up to 3 GHz, an Agilent high-frequency analyzer (model E4991 A) was connected to a Novocontrol high-frequency cell.^56–59^ The sample was exposed to temperatures ranging from 173 to 473 K, controlled by a QUATRO cryosystem (Novocontrol) operated with liquid N_2_. The system controls the temperature by limiting gas flow and heater power simultaneously. Experimental details and the exact setup have already been described elsewhere.^56–59^

## Results and Discussion

The transients obtained from variable-temperature ^7^Li NMR measurements in the laboratory and rotating frames are shown in Figure 2. The corresponding build-up curves (*R*_1_) and decay curves (*R*_1ρ_) are normalized to the interval [0,1] to facilitate comparison.

**Figure 2:**
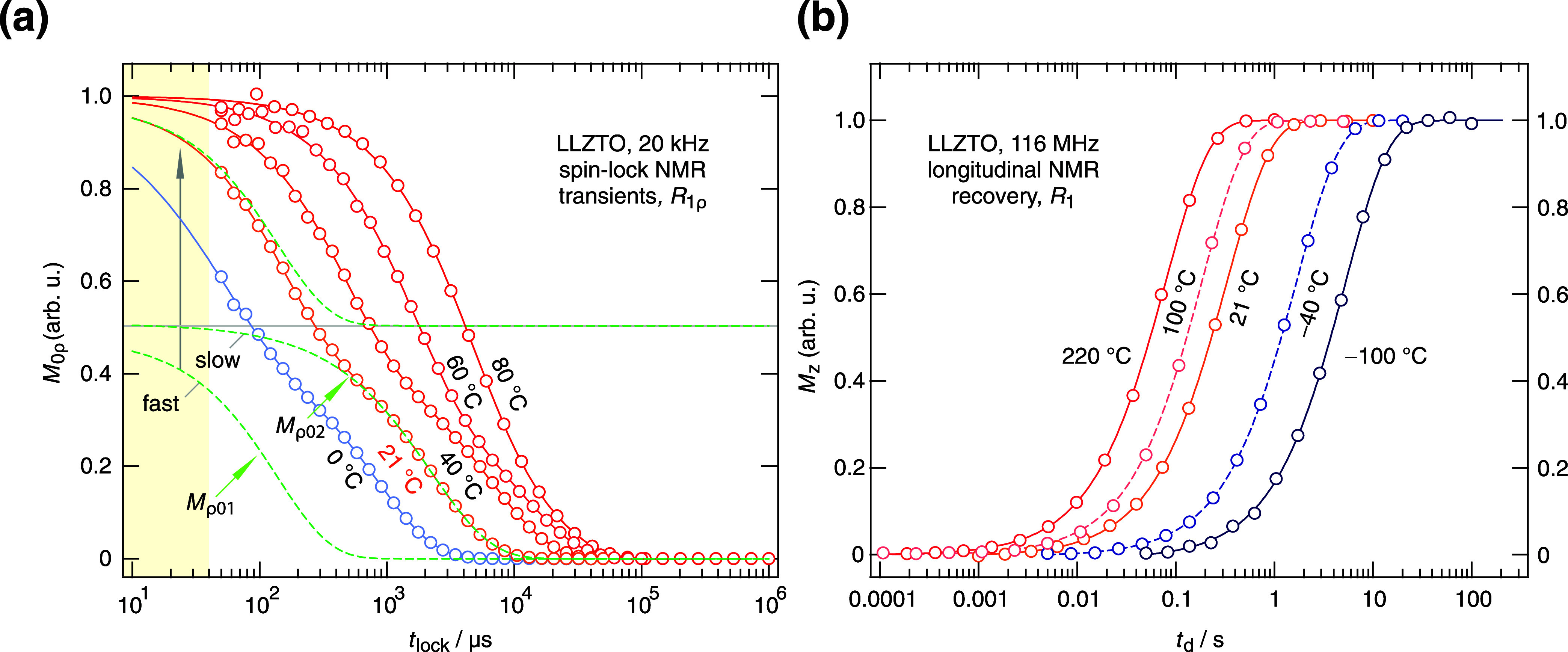
^7^Li NMR magnetization transients *M*_ρ_(*t*_lock_) and *M*_*z*_(*t*_d_) recorded for single crystalline LLZTO at a Larmor frequency of 116 MHz and a locking frequency of 20 kHz. In (a) the spin-lock curves are shown which adopt biexponential behavior at higher temperatures. For the transient referring to 21 °C, the two individual (slightly stretched) exponentials are shown as dashed lines. They are labeled *M*_ρ01_ and *M*_ρ02_ mirroring the fast and slowly relaxing components. Data points in the locking regime shorter than 40 μs are less accurately accessible. (b) *R*_1_^7^Li NMR transients of LLZTO that follow single exponential recovery. Data shown are representative of the full temperature range; transients (*M*_*z*_ and *M*_ρ_) at other temperatures exhibit comparable quality and behavior.

The transients associated with *R*_1_ are well described by single stretched exponential functions (see Figure 2b). The resulting rates are displayed in the Arrhenius representation in Figure 3. Upon increasing temperature *T*, the rates evolves from a low-temperature background into a diffusion-induced relaxation-rate peak *R*_1_(1/*T*) centered at *T*_max_ = 392 K. At this temperature the motional correlation time reaches the (maximum) condition ω_0_τ_c_ ≈ 1. To evaluate the shape of the ^7^Li NMR rate peak and the background contribution observed at lower temperatures, we employed the Bloembergen-Purcell-Pound (BPP) formalism,^60–62^ incorporating an asymmetry parameter β as well as a power-law term, *R*_1_ ∼ *T* ^*k*^, to account for nondiffusive background contributions.^63^

1 $$R_{1}∼J\left(\omega_{0}\right)=C\frac{\tau_{c}}{1+{\left(\omega_{0}\tau_{c}\right)}^{\beta }}+aT^{k}$$

The motional correlation rate in (eq 1), which is of the same order of magnitude as the Li^+^ jump rate τ^–1^, is assumed to follow an Arrhenius temperature dependence; *k*_B_ denotes Boltzmann’s constant.

2 $${\tau_{c}}^{-1}\approx \tau^{-1}=\tau_{0\left(c\right)}^{-1}\exp\left(-E_{a}/\left(k_{B}T\right)\right)$$

**Figure 3:**
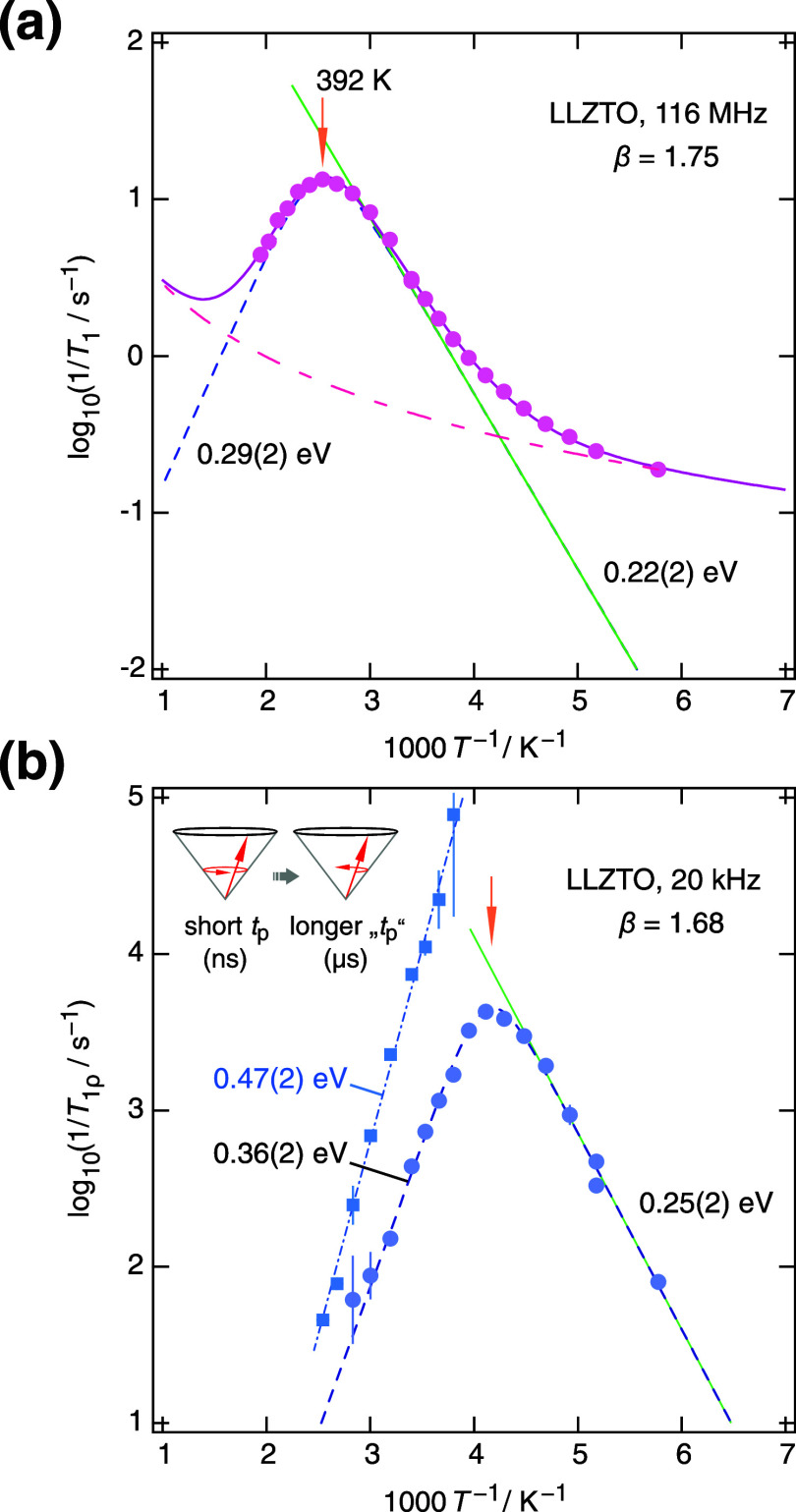
Arrhenius plots illustrating the temperature dependence of the (a) longitudinal (1/*T*_1_) and the (b) spin-lock ^7^Li NMR spin–lattice relaxation rate (1/*T*_1ρ_) in single-crystalline LLZTO. (a) The solid line represents the overall fit, including the onset of nondiffusive background relaxation at low temperatures. This background contribution, approximated by a power law, *T*_1_ ∼ *T* ^1.57^, is shown as a dashed line. The fine dashed line denotes the Lorentzian-shaped purely diffusion-induced contribution, described by an asymmetric BPP-type spectral density with β = 1.75(5), which yields a reduced low-temperature slope corresponding to 0.22(2) eV in the regime ω_0_τ ≫ 1. The activation energy extracted from the BPP-fit (see eq 1) amounts to 0.29(2) eV. This value is governing the slope of the high-*T* flank of the peak. (b) The dashed line represents the spin-lock (single-term) BPP fit to evaluate the shape of the response in the rotating frame of reference (20 kHz spin-lock frequency; 116 MHz). Again, an asymmetric (diffusion-induced) peak is obtained yielding 0.36(2) eV and 0.25(2) eV. The value of 0.36 eV characterizes long-range ion transport in LLZTO. In addition, the spin-lock experiments reveal a faster (transversal) decay process whose rates obey Arrhenius behavior with an activation energy of 0.47(2) eV, which aligns well with the activation energy from DC conductivity measurements (0.41(1) to 0.44(1) eV) probing macroscopic Li^+^ ion transport, see below. Vertical lines represent error bars corresponding to the standard deviation derived from analysis of the underlying transients. Error bars for *R*_1_ are equal to or smaller than the symbol size.

The fit (eq 1) shown in Figure 3, performed in log-space, yields an Arrhenius prefactor of τ_0_ = 2.5(5) × 10^–13^ s, which lies in the range of phonon frequencies. The corresponding activation energy, *E*_a_, amounts to 0.29(2) eV and is attributed to a combination of long-range (and short-range) Li^+^ motional processes, involving jumps between the 96*h* and 24*d* sites.^43,64^ This value characterizes the high-temperature flank of the BPP-type relaxation peak.^65^ On the low-temperature side, the flank is governed by a lower activation energy of 0.22(2) eV, indicating a stronger influence of short-range, localized spin fluctuations controlling nuclear spin relaxation.^65^ Hence, the ^7^Li NMR *R*_1_ experiments already point to frequency-dependent Li^+^ ion dynamics^58,66^ in LLZTO, as the relaxation-rate peak recorded in the MHz regime is characterized by two distinct activation energies. Whereas the higher value of 0.29 eV (Figure 3a) reflects contributions from long-range ion transport, the lower value of 0.22 eV is governed by local motional processes associated with Coulomb interactions and structural point disorder in LLZTO. The combined effect of these two factors leads to correlated ionic motion and has been shown to give rise to asymmetric BPP-type relaxation-rate peaks.^67^

In NMR relaxation, the characteristic time window of the experiment is set by the period of one full Larmor precession, *t*_p_ = 1/ω_0_ = 1.37 × 10^–9^ s (≈ 1.4 ns for ω_0_/2π = 116 MHz). At low temperatures, only a small number of (jump) events occur within this ns time interval. Consequently, the spin fluctuations contributing to *R*_1_ predominantly arise from highly probable, low-energy local motions associated with potential minima connected by low energy barriers.^65^ As the temperature increases, the number of motional events occurring within *t*_p_ grows, and the likelihood that jumps over higher energy barriers are sampled correspondingly increases. As a result, the apparent activation energy, associated with the limit ω_0_τ ≫ 1, rises when going from the low- to the high-temperature regime, for which ω_0_τ ≪ 1 holds. Thus, NMR becomes sensitive to short-range ion dynamics at low temperatures and progressively to long-range ion transport at higher temperatures, purely due to the temperature-dependent change in the experimental selectivity.^65^ This change is captured by the parameter β, for which values β < 2, here β ≈ 7/4, are obtained in the case of correlated ion diffusion (see above). For a sample exhibiting a fully homogeneous (or uniform) potential landscape, i.e., with no distinction between short- and long-range motion and characterized by a single dominant energy barrier, a symmetric relaxation-rate peak is expected (β = 2).^68^

Increasing the time window *t*_p_ is expected to enhance the sensitivity of the experiment, making it more capable of capturing dipolar and quadrupolar spin fluctuations associated with long-range Li^+^ ion dynamics. An increase in *t*_p_ from the nanosecond to the low-microsecond regime can be achieved by performing spin-lock NMR experiments in the rotating frame,^69^ where *t*_p_ = 1/ω_1_, i.e., determined by the angular locking frequency. For a typical (technical) locking field of ω_1_ = 1.25 × 10^5^ rad s^–1^, this yields *t*_p_ = 7.95 × 10^–6^ s (8 μs).

The diffusion-induced spin-lock NMR *R*_1ρ_ rates are shown in Figure 3b. Above 250 K, the magnetization transients exhibit a clear two-step decay, which we model as a sum of two stretched exponentials. At lower temperatures, however, the transients are well described by a single stretched-exponential function. Although fits using two stretched exponentials were also attempted over the full temperature range, no statistically significant improvement was obtained below approximately 250 K, indicating that the second relaxation process only emerges at elevated temperatures. The slower relaxation component follows a BPP-type response, whereas the faster component displays a high-temperature flank with an activation energy of 0.47(2) eV. This value is in excellent agreement with the activation energy obtained from DC conductivity (0.41(1) to 0.44(1) eV, see below), which probes macroscopic Li^+^ transport. The slower rates are well described by a single-term, asymmetric spin-lock BPP peak, *R*_1ρ_ ∼ τ_c_/(1 + (2ω_1_τ_c_)^β^), whose asymmetry parameter (β = 1.68(5)) matches that of the *R*_1_ BPP peak (β = 1.75(5), see Figure 3a). The BPP activation energy for this component is 0.36(2) eV (τ_0_ = 2(1) × 10^–13^ s), which we assign to long-range ion transport in LLZTO. In contrast, the lower apparent value of 0.25(2) eV extracted from the linear low-temperature regime is likely still influenced by short-range motions. Overall, the NMR relaxation data indicate activation energies spanning 0.22(2) to 0.47(2) eV, depending on the sensitivity window of the experiment and the specific spin fluctuations being probed.

To go beyond analyzing only the two spin-lock relaxation rates, we also evaluated the individual amplitudes *M*_ρ01_ and *M*_ρ02_ of the two decay components. These amplitudes are plotted in Figure 4 as a function of inverse temperature (1/*T*) to enable direct comparison with the evolution of the NSR rates shown in Figure 3b. It is evident that the faster relaxation component progressively dominates the overall transients with increasing temperature. This process, characterized by an activation energy of 0.47(2) eV, is expected to govern long-range ion transport in LLZTO. Consistent with this interpretation, the conductivity spectroscopy presented below points to the same activation energy. As the two relaxation processes exhibit distinctly different temperature dependences in the high-temperature regime where ω_1_τ ≪ 1 holds, we therefore conclude that they reflect two separate motional processes detected by NMR, rather than arising from quadrupolar relaxation effects as suggested and recently revisited by Wimperis et al.^70^ for *R*_1_ measurements in other fast ionic conductors.

**Figure 4:**
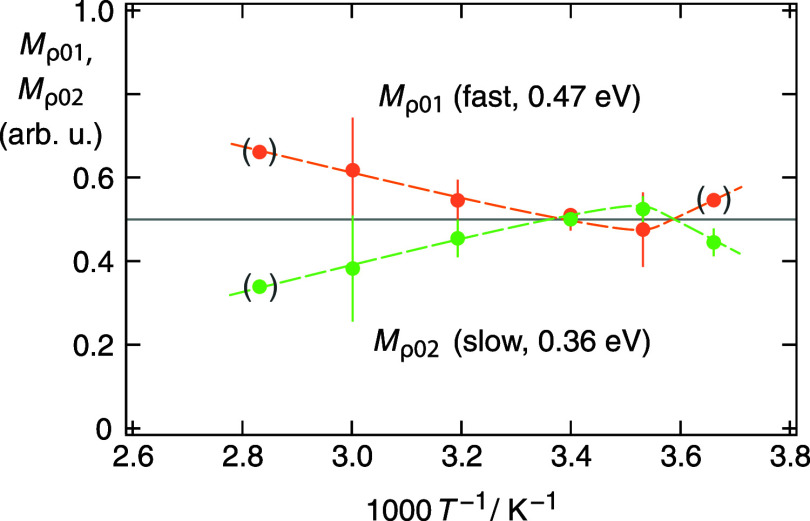
^7^Li NMR spin-lock magnetization transients of LLZTO are parametrized with a sum of two exponential functions for *T* > 250 K; the associated (normalized) amplitudes *M*_ρ01_ and *M*_ρ02_ of the fast and slowly decaying contributions (see Figure 2a) are shown as a function of the inverse temperature. With increasing temperature, the transients are increasingly governed by the faster process characterized by *M*_ρ01_, which mirrors a dynamic process activated by 0.47 eV, see Figure 3b. Values in round brackets show large errors and serve as estimations only.

Finally, to obtain a comprehensive description of diffusion-controlled ^7^Li NMR relaxation in LLZTO, we tried to analyze the *R*_1_ and *R*_1ρ_ data using a single set of parameters within a so-called joint analysis. The resulting global fit, in which the *E*_a_ fittings parameters are linked, is shown in Figure 5. For comparison, data for single-crystalline Li_6_La_3_ZrTaO_12_ are included as well. The solid line represents the global fit employing a two-term BPP expression for *R*_1_ and a three-term BPP expression for *R*_1ρ_, see (eq 3) with τ_c1_ and τ_c1ρ_ denoting the motional correlation times in the laboratory and rotating frame of reference, respectively.^61,62^

3 $$R_{1}=C_{1}\left[\frac{\tau_{c1}}{1+{\left(\omega_{0}\tau_{c1}\right)}^{2}}+\frac{4\tau_{c1}}{1+{\left(2\omega_{0}\tau_{c1}\right)}^{2}}\right]\;\mathrm{and}\;R_{1\rho }=C_{1\rho }\left[\frac{6\tau_{c1\rho }}{1+{\left(2\omega_{1}\tau_{c1\rho }\right)}^{2}}+\frac{10\tau_{c1\rho }}{1+{\left(\omega_{0}\tau_{c1\rho }\right)}^{2}}+\frac{4\tau_{c1\rho }}{1+{\left(2\omega_{0}\tau_{c1\rho }\right)}^{2}}\right]$$

**Figure 5:**
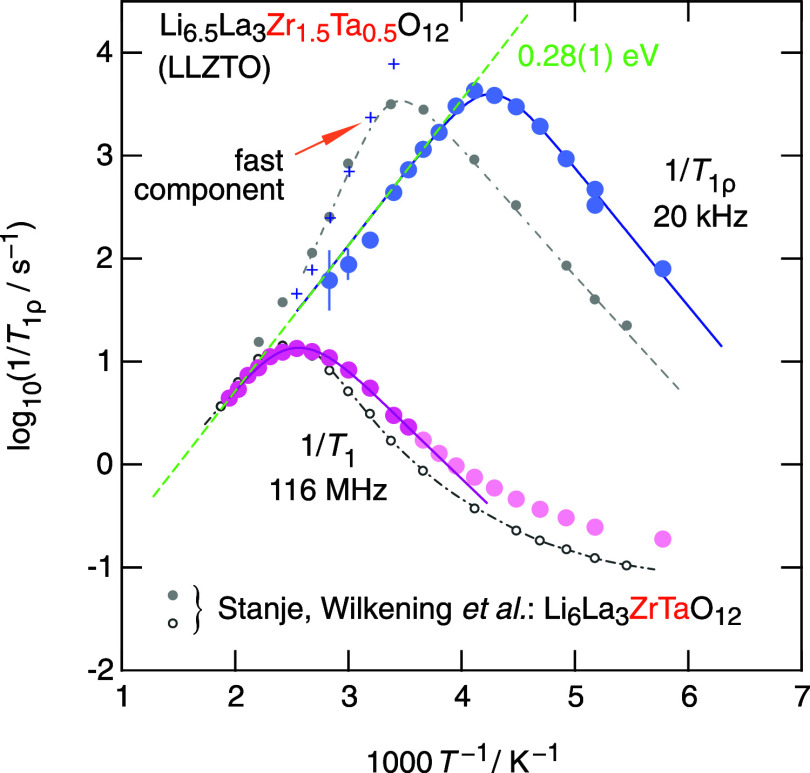
Global relaxation analysis of the ^7^Li NMR relaxation response in LLZTO across the kHz and MHz frequency regimes, modeled with the same activation energy *E*_a_ determining the slope on the high-*T* flanks (as indicated by the dashed line) but with individual Arrhenius prefactors (τ_0_, see eq 2) governing the two rate peaks. For comparison, the corresponding spin-lock and laboratory-frame NSR rates of single-crystalline Li_6_La_3_ZrTaO_12_ with a Zr:Ta ratio of 1:1 are shown for comparison. Crosses indicate the fast relaxation rates from spin-lock NMR conducted at higher temperatures. See text for further explanation. Vertical lines represent error bars corresponding to the standard deviation derived from analysis of the underlying transients; they are omitted for the fast-decaying component but shown in Figure 3.

The global fit was constructed so that the same activation energy governs the high-temperature flanks of both relaxation-rate peaks. However, also constraining the pre-exponential factor does not yield an acceptable fit, consistent with the findings of Stanje et al.^48^ for Li_6_La_3_ZrTaO_12_, where a global fit turned out to be successful because the spin-lock rates were not biexponential. In our case, the pronounced biexponentiality precludes a robust global fit linking both *E*_a_ and τ_0_; instead, we report two separate prefactors, namely τ_c01_ (ca. 2.4 × 10^–13^ s) and τ_c01ρ_ (ca. 3.7 × 10^–12^ s). Ideally, a model with a constrained prefactor τ_c0_ should capture the crossover between rotating-frame (spin-lock) and laboratory-frame relaxation, as represented by the multiterm fitting function (eq 3). Here, however, the crossover region is obscured by the biexponential decay. Although the fit in Figure 5 appears reasonable at first glance, it does not accurately reproduce the transition regime and therefore returns an activation energy (0.28(1) eV) that is systematically lower than the value obtained from the separate analysis of the spin-lock data.

Interestingly, the faster spin-lock relaxation rates shown in Figure 3b nearly coincide with the high-temperature flank of the *R*_1ρ_ peak reported by Stanje et al. for Li_6_La_3_ZrTaO_12_.^48^ For direct comparison, those literature data are included in Figure 5 and plotted as crosses. In single-crystalline Li_6_La_3_ZrTaO_12_, long-range ion dynamics is governed by an activation energy of 0.51 eV,^48^ slightly larger than in the present case (0.47(2) eV). Although the apparent shift of the spin-lock rate peak, produced by the slower relaxation process, toward lower temperatures might suggest enhanced (long-range) ion dynamics in LLZTO with a Zr:Ta ratio of 1.5:0.5, the pronounced biexponentiality of the spin-lock transients—particularly the fast *R*_1ρ_ component coinciding with the literature data (Zr:Ta, 1:1)—indicates that the underlying ion dynamics are very similar. Conversely, the nearly identical activation energies, combined with the larger NSR pre-exponential factor (10^15^ s^–1^) observed for single-crystalline Li_6_La_3_ZrTaO_12_, may even indicate faster ion dynamics in the crystal with a Zr:Ta ratio of 1:1 investigated earlier. Information from electrical modulus spectroscopy is helpful in informing this discussion, as outlined below.

To quantify ionic transport and compare the conductivities of the two crystals with different Zr:Ta ratios, we performed impedance spectroscopy and analyzed both conductivity isotherms and electric-modulus peaks. Conductivity isotherms from −100 to 200 °C are shown in Figure 6a, combining data from a standard cell (up to 10^7^ Hz) with measurements using a high-frequency (HF) cell capable of capturing the response up to 3 × 10^9^ Hz (open symbols). Careful cell calibration is required to avoid systematic offsets; in our case, the two data sets agree well, with no significant vertical offset.

**Figure 6:**
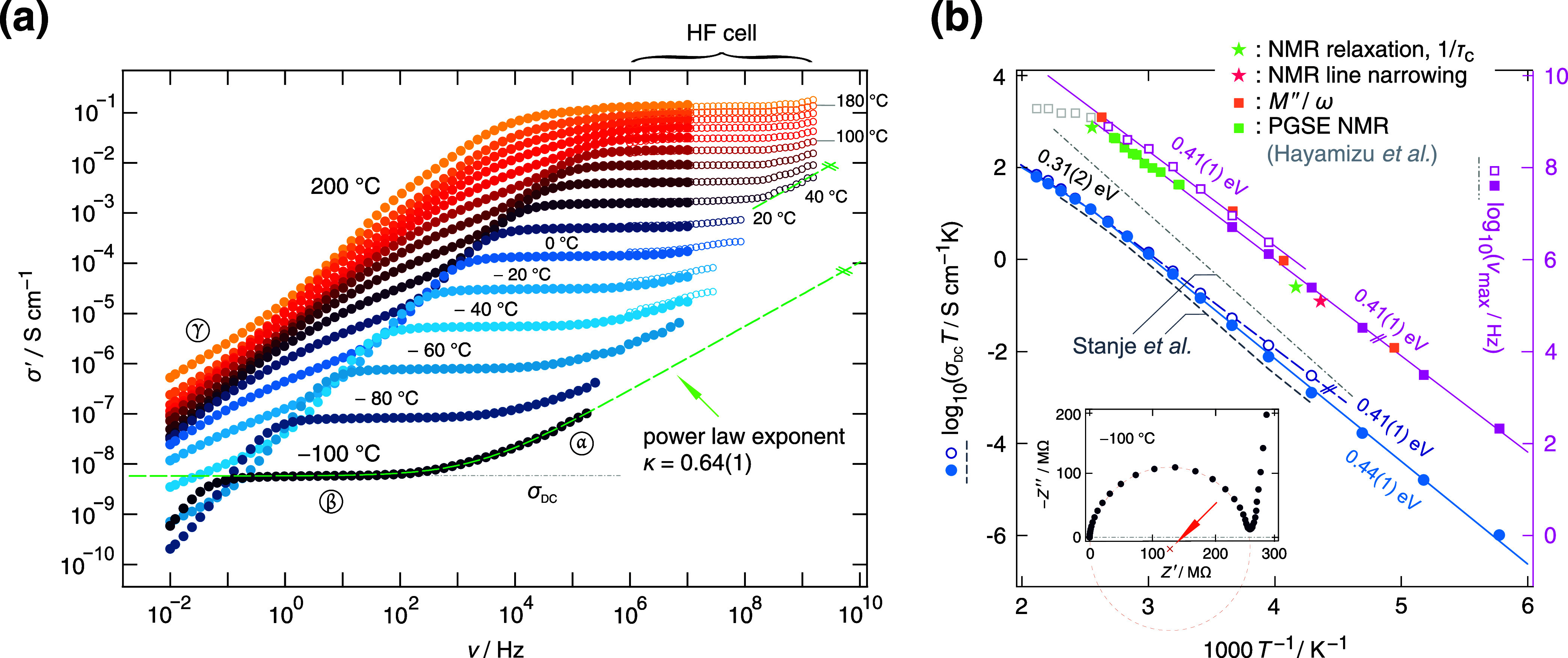
(a) Conductivity isotherms recorded over a broad frequency range to capture both long-range and short-range ion dynamics in LLZTO. Short-range (correlated) motion manifests as a dispersive high-frequency response (α), most pronounced at low temperatures. The prominent dc plateaus (β) are used to extract the long-range ionic conductivity analyzed in (b); at 293 K, the conductivity is 5 × 10^–4^ S cm^–1^. (b) Arrhenius plot of σ_dc_ versus inverse temperature 1/*T*. High-frequency (HF) measurements (open symbols) yield a long-range activation energy of *E*_a_ = 0.41(1) eV, while data from the standard cell show a slightly higher value of 0.44(1) eV. Both Arrhenius trends deviate at higher temperatures, entering a regime characterized by 0.31(2) eV. Similar behavior has been reported for LLZTO with a Zr:Ta ratio of 1:1.^48^ Dashed (σ_dc_ *T*) and dashed-dotted (ν_max_) lines refer to results from Stanje et al.^48^ For comparison, characteristic electrical relaxation frequencies from modulus spectroscopy (right axis; again, open symbols refer to the HF cell) are included and are consistent with 0.41(1) eV; see text for details. Jump rates from ^7^Li NMR (*R*_1_ and *R*_1ρ_), line narrowing, and resistivity measurements (*M*″/ω) are included as well together with results from field gradient NMR reported by Hayamizu et al.^47^ The inset displays the complex-plane (Nyquist) plot at – 100 °C, highlighting the semicircular arc associated with the corresponding isotherm shown in (a). The arrow points to the center of the semicircle which almost coincides with the real axis.

The isotherms in Figure 6a can be divided into several regions. At high frequencies, a dispersive regime (α regime) is observed that reflects correlated motion, including forward–backward jumps that do not contribute to long-range transport. This dispersive part (see arrow in Figure 6a) follows a Jonscher-type^71^ power law, σ′ ∝ ν^κ^, with κ = 0.64 at −100 °C (represented as a dashed line), a typical value for such correlation effects. At lower frequencies, the curves merge into a frequency-independent plateau (β regime) associated with bulk long-range ionic transport (dc conductivity, σ_dc_). This plateau is also shown in Figure 7c for the isotherm recorded at −20 °C. The corresponding capacitance is on the order of a few pF. This capacitance value is either obtained directly from the complex-plane impedance plot (see inset in Figure 6b) or from analyzing the capacitance *C*′ as a function of frequency. Values in the pF range correspond to relative electrical permittivities *ε*′ of ca. 66 at −20 °C and approximately 42 at 80 °C, see Figure 7b and its inset. For comparison, in Figure 7b,7c the respective isotherms of the imaginary parts of the complex permittivity and conductivity, ε″ and σ′′, are also shown. Coming back to the Nyquist representation in Figure 6b, we recognize that the nearly perfect semicircle, with its center slightly below the *Z*′ axis (see arrow in Figure 6b, inset), is primarily governed by the broad dc conductivity plateau. Electrode polarization produces the low-frequency spike. In the transition region, a minor semicircle attributable to surface-related processes could explain why the data do not intersect the *Z*′-axis perfectly. This effect is especially evident at higher temperatures, where these features shift into the high-frequency window. The same features are seen below the dc plateaus of the conductivity isotherms shown in Figure 6a; they transition into curved, frequency-dependent regions (γ regime, see Figures 6a and 7c) that first reflect near-surface electrical relaxation processes, likely influenced by residual carbonate on the LLZTO surface, and, more prominently, electrode polarization effects that cause the above-mentioned charge accumulation at the ion-blocking metallic contacts. The same features are also seen in electrical permittivity isotherms ε′(ν), see Figure 7b. At frequencies below 100 Hz, the permittivity is dominated by polarization and interfacial effects.

**Figure 7:**
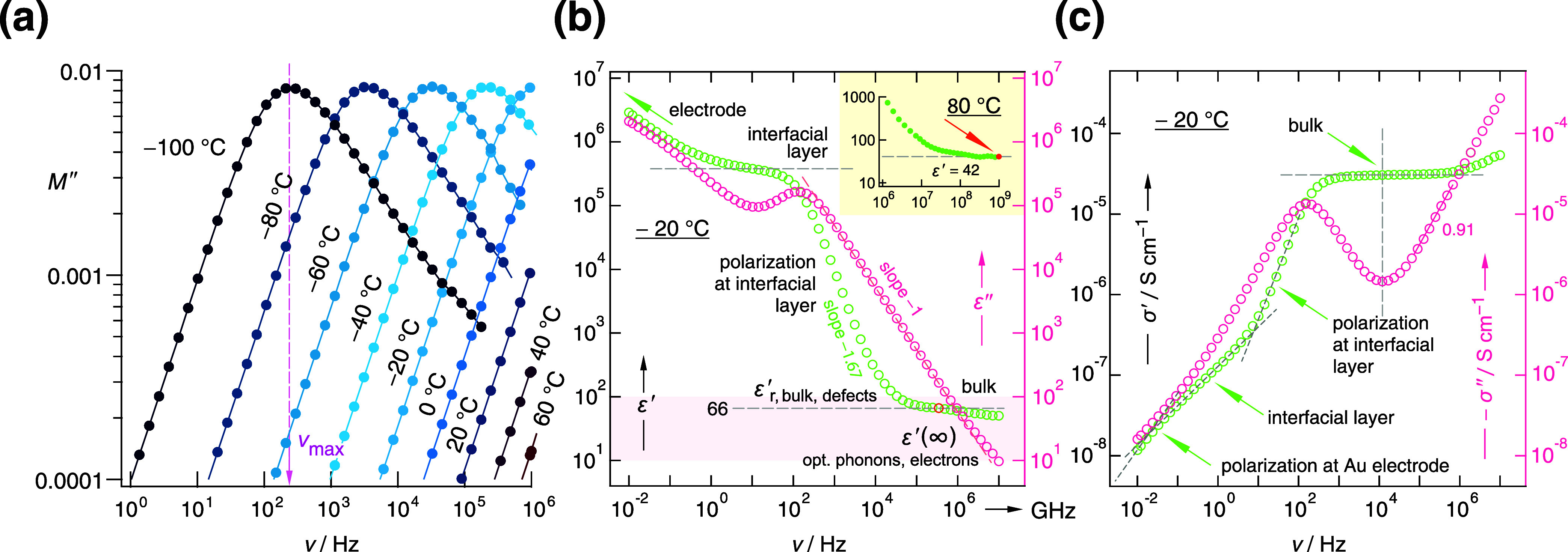
(a) Electrical modulus spectra recorded at the temperatures indicated. The peak position on the frequency axis defines the characteristic electrical relaxation frequency (shown for the curve recorded at −100 °C) analyzed in Figure 6b. (b) Permittivity isotherms measured at −20 °C and at 80 °C (inset). Plotting the real part of the complex permittivity versus frequency shows the same features as identified when analyzing σ′(ν). Ionic, relative permittivities referring to bulk properties range from 66 to 42 (inset) when going from −20 to 80 °C. (c) Comparison of σ′(ν) with σ″(ν); from the prominent bulk plateau of σ′(ν) the conductivity can be accurately determined. A stepwise polarization decay shows electrode polarization presumably combined with polarization at interfacial layers.

The Arrhenius plot (σ_dc_ *T* vs 1/*T*) in Figure 6b was used to extract the long-range activation energies for Li^+^ transport in LZTO. The two setups yield activation energies ranging from 0.41(1) to 0.44(1) eV, the latter in excellent agreement with previously reported values of 0.43 and 0.44 eV.^30,72^ A value of 0.43 eV has also been derived from field gradient NMR.^72^ Hayamizu et al. report a value of 0.39 eV from pulsed gradient spin–echo NMR.^47^ At 293 K, the dc conductivity was 0.5 × 10^–3^ S cm^–1^, which is consistent with previously reported values of 1.27 × 10^–3^ S cm^–1^ and 0.7 × 10^–3^ S cm^–1^ on single crystals of the same composition.^30,72^

To compare the activation energies directly derived from the dc plateaus, we also analyzed the characteristic electrical relaxation frequencies ν_max_ from modulus spectra *M*″(ν) shown in Figure 7a. For both measurement cells, the high-frequency and the standard cell, only a minor offset is observed in the Arrhenius plot (Figure 6b; while open symbols refer to the high-frequency cell, closed ones represent results obtained with the standard cell setup). Both data sets yield an activation energy of *E*_a_ = 0.41(1) eV, in excellent agreement with the values obtained from the dc plateaus. A slight apparent decrease in *E*_a_ from the modulus analysis can be explained by a weak temperature dependence of the charge-carrier concentration *N*_c_, which increases smoothly with temperature; identical values would imply a temperature-invariant carrier concentration. Data points at the highest temperatures (gray symbols) deviate from the Arrhenius trend, likely due to measurement inaccuracies at very high frequencies.

A further link between NMR and electric modulus data emerges when the so-called resistivity *M*″/ω is analyzed as a function of temperature at fixed frequencies ν = ω/2π.^58,73^ The corresponding plot is shown in Figure 8. The resulting BPP-like peaks are asymmetric, with coinciding flanks on the high-temperature side that yield an activation energy of 0.44(1) eV. This value also reflects the activation energy obtained from dc-conductivity spectroscopy. In contrast, the low-temperature side is characterized by a smaller apparent activation energy, reflecting short-range ion dynamics. The value of 0.20(2) eV is in excellent agreement with that obtained from *R*_1_^7^Li NSR (see above). Deviations observed at 1.23 GHz originate from technical limitations of this analysis; therefore, the value of 0.15 eV should be treated with caution.

**Figure 8:**
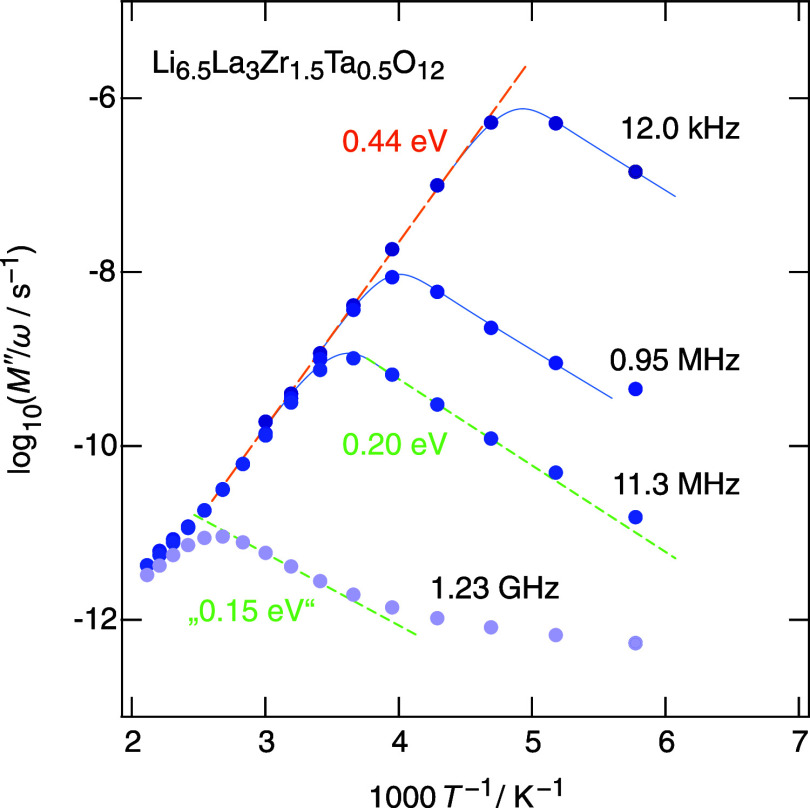
Resistivity values, defined as *M*″/ω, plotted as a function of inverse temperature at constant frequencies. The asymmetric peaks of single crystalline Li_6.5_La_3_Zr_1.5_Ta_0.5_O_12_ reveal activation energies of 0.44(1) eV and 0.20(2) eV, as indicated. The peak positions lead to the jump rates shown in Figure 6b along with those derived from diffusion-induced ^7^Li NSR NMR and electrical modulus spectroscopy, see Figure 7a.

In summary, long-range ionic transport in single-crystalline LLZTO with a Zr:Ta ratio of 1.5:0.5 is characterized by an activation energy of 0.41(1) to 0.44(1) eV. The value of 0.44 eV is close to that inferred from the fast spin-lock NMR relaxation rates (0.47(2) eV), whereas 0.41 eV closely matches the average of the activation energies sensed by the fast and slow components of the spin-lock transients (0.47(2) eV and 0.36(2) eV; see Figure 3b). Together, these results provide a consistent picture of ion dynamics from two complementary probes of nuclear spin fluctuations and electrical relaxation. Notably, the value of 0.31(2) eV observed in dc conductivity at higher temperatures matches the activation energy obtained from laboratory-frame NMR on the high-*T* side, when that peak is analyzed separately with an appropriate nondiffusive background contribution (Figure 3a). In addition, activation energies as low as 0.2 eV, characterizing short-range, localized ionic jump processes, are obtained from *R*_1_^7^Li NSR in the low-temperature regime and from resistivity analyses (see Figure 8), likewise at lower temperatures.

To directly compare motional correlation rates quantitatively, we estimated the corresponding rates from the NMR peak maxima, using the maximum conditions ω_0_τ_c_ ≈ 1 and ω_1_τ_c_ ≈ 0.5, and included them in Figure 6b (stars). The two data points align with the activation energy derived from electrical measurements and support the modulus-based analysis quantitatively. Since the correlation rate from spin-lock NMR captures only a subset of ion motions, it is expected to be somewhat lower than the rate inferred from modulus spectroscopy. By contrast, the rate deduced from diffusion-controlled laboratory-frame (*R*_1_) NMR lies close to the Arrhenius trend from electric modulus spectroscopy, indicating that *R*_1_ NMR, at least at *T*_max_, is already substantially influenced by jump processes that contribute to long-range ionic transport, and not solely by short-range dynamics, as is sometimes erroneously propagated. Finally, the position of each *M*″/ω curve in Figure 8 defines a relaxation rate that can be also interpreted as being close to the ionic jump rate. In the Arrhenius plot shown in Figure 6b, these additional rates have been included and they complement the Arrhenius lines derived from the *M*″ peak maxima (see Figure 7a) very well.

^7^Li NMR line shapes can also be used to further complement the Arrhenius analysis. In Figure 9, we plot the motion-induced narrowing of the ^7^Li NMR central transition of the spin-3/2 nuclei. The solid line represents a fit based on Abragam’s formula,^74^ discussed elsewhere.^63^ The resulting implicit functional relationship between the line width (full width at half-maximum, FWHM) and temperature requires a dedicated numerical procedure to obtain the solution. The fit yields an activation energy of 0.22(2) eV. Although this value is in good agreement with the activation energy derived for short-range Li hopping from *R*_1_, the Abragam analysis is known to typically underestimate activation energies.^75^ A more reliable indicator of jump diffusion can be obtained from the inflection point of the narrowing curve. From this point, we estimate a jump rate on the order of 8 kHz × 2π. A rigid-lattice line width of approximately 8 kHz has also been observed for Li_6_La_3_ZrTaO_12_. The corresponding jump rate is included in Figure 6b and is in excellent agreement with the jump rates estimated from NSR and modulus spectroscopy representing macroscopic Li^+^ diffusion in LLZTO.

**Figure 9:**
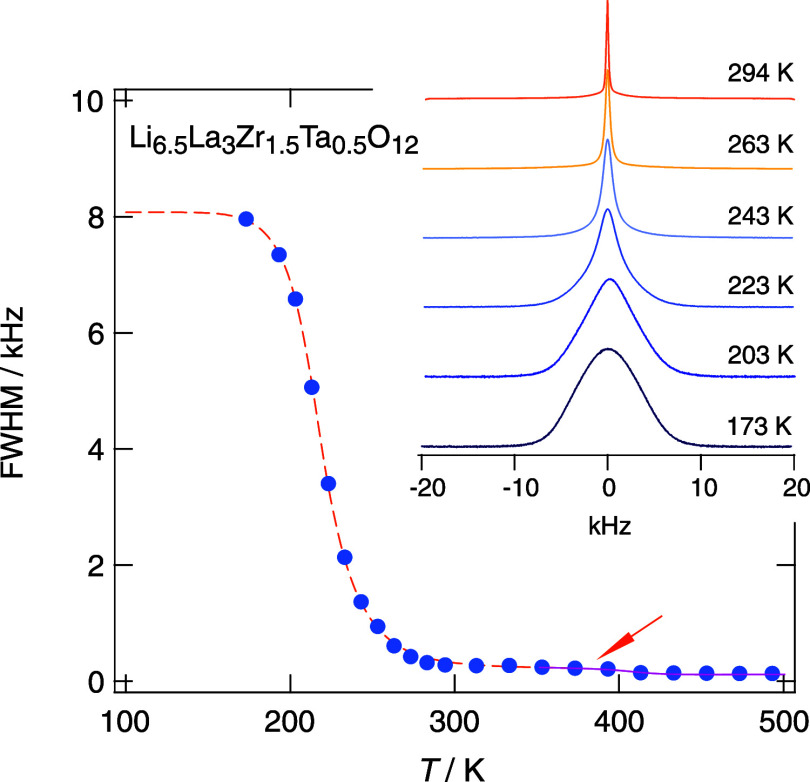
Motional narrowing of the ^7^Li NMR central transition with increasing temperature. The progressive reduction in line width is governed by averaging of dipole–dipole interactions induced by thermally activated Li^+^ hopping processes. Full width at half-maximum (FWHM) values were read off directly from the ^7^Li NMR spectra shown in the inset. The dashed line represents a fit based on the Abragam model (see text) of motional line narrowing, yielding an activation energy of 0.22(2) eV. The inflection point of the curve at 218 K corresponds to a jump rate of approximately 8 kHz × 2π. Except for the line recorded at 223 K, which may consist of a superposition of a narrow and a broader component, the spectra do not clearly reflect the heterogeneous dynamics suggested by the *R*_1_ NSR results. A very shallow additional decay step, starting at 360 K and indicated by the arrow, may reflect a very small subset of Li ions that undergo motional narrowing only at much higher temperatures. This contribution was treated as negligible.

An additional level of agreement is achieved when the recent jump diffusion coefficients *D* for single crystalline Li_6.5_La_3_Zr_1.5_Ta_0.5_O_12_ reported by Hayamizu et al.^47^ using pulsed-gradient spin–echo (PGSE) NMR are included in Figure 6b. We have converted these values with the Einstein-Smuoluchowski equation into jump rates or correlation rates 1/τ_c_ according to 1/τ_c_ = 6*D*/*a* ^2^; for the jump distance we estimated a mean value of 2 Å. These values align closely with both the electrical and NMR measurements of the present study. For better comparison, the same data from Figure 6b are also shown in Figure 10a. In addition, in Figure 10a, also the diffusion coefficients reported by Kataoka and Akimoto^30^ have been included as 1/τ_c_.

**Figure 10:**
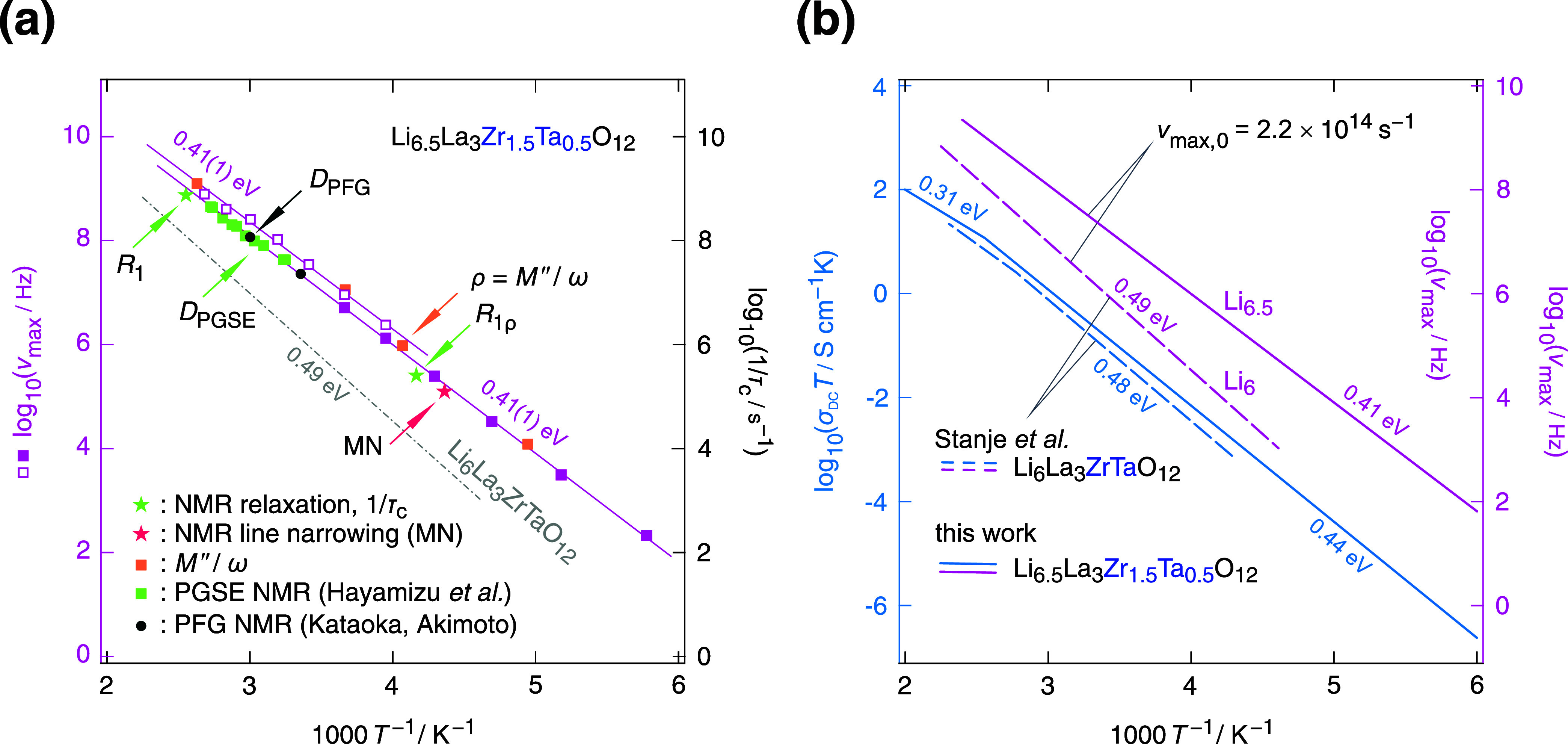
(a) Comparison of ν_max_ for Li_6.5_La_3_Zr_1.5_Ta_0.5_O_12_ from Figure 6b with values obtained from ^7^Li NMR relaxation, motional line narrowing (MN), electrical resistivity, and (pulsed) field-gradient NMR measurements reported by Hayamizu et al.^47^ as well as by Kataoka and Akimoto.^30^ See text for further details. (b) Comparing (bulk) dc conductivities and hopping rates, deduced from electric modulus spectroscopy, for the two LLZTO single crystals with different Li contents adjusted via the Zr:Ta ratio. Li_6.5_La_3_Zr_1.5_Ta_0.5_O_12_ reveals a clearly higher hopping frequency ν_max_. This finding does, however, not translate into a conductivity higher by the same order of magnitude, presumably due to a reduced effective charge-carrier concentration *N*_c_ caused by Li “stuffing”. In Li_6_La_3_ZrTaO_12_ a higher *N*_c_ compensates for the lower Li^+^ mobility, yielding relatively good ionic conductivity. It is important to note that the prefactor of the ν_max_(1/*T*) Arrhenius lines is the same for both compounds (2.2(5) × 10^14^ s^–1^), so the difference between the two Arrhenius lines is determined solely by the difference in activation energy (0.49 eV vs 0.41 eV), as expected if *N*_c_ should play a key role in explaining the difference between the two systems. The factor of 10^14^ s^–1^ is higher than that seen in NSR for Li_6.5_La_3_Zr_1.5_Ta_0.5_O_12_ (2.5 × 10^13^ s^–1^, see above), but lower than that derived from NMR relaxation of Li_6_La_3_ZrTaO_12_ by Stanje et al. (1.5 × 10^15^ s^–1^).^48^

Taken together, and disregarding the small offset seen in the Arrhenius lines governed by 0.41(1) eV in Figure 6b, the data indicate that an activation energy of about 0.41(1) eV characterizes long-range ion dynamics in Li_6.5_La_3_Zr_1.5_Ta_0.5_O_12_. This conclusion is based on a comprehensive kHz to GHz analysis combining results from nuclear resonance and electrical measurements (modulus and resistivity data). For comparison, jump rates obtained from electrical modulus spectroscopy on Li_6_La_3_ZrTaO_12_, reported earlier by some of us,^48^ are also indicated (as dashed-dotted line) in Figure 6b and in Figure 10a. We clearly see that, although both single crystals exhibit almost the same dc conductivities (5 × 10^–4^ S cm^–1^ (Li6.5 phase) and 3 × 10^–4^ S cm^–1^ (Li6 phase)^48^ at 293 K), the electrical relaxation rates of Li_6.5_La_3_Zr_1.5_Ta_0.5_O_12_ are higher by approximately 1 order of magnitude than those in Li_6_La_3_ZrTaO_12_ (see Figures 6b and 10a). Consistently, the activation energy increases from 0.41 to 0.49 eV, see Stanje et al.,^48^ with increasing Ta content. This observation is clarified in Figure 10 including the corresponding Arrhenius lines from the two studies for better comparison.

The observed change in mobility, i.e., in hopping frequency ν_max_, at nearly constant conductivity (σ_dc_ *T*, see the comparison in Figure 10b) can be explained by a variation in the effective number of charge carriers, *N*_c_, which appears to be higher in Li_6_La_3_ZrTaO_12_ and thus compensates for the lower intrinsic mobility of the Li^+^ ions given that σ_dc_ ∼ *N*_c_ and ν_max_ is not a function of *N*_c_. This view is supported by the observation that the prefactor ν_max, 0_ of the ν_max_(1/*T*) Arrhenius lines in Figure 10b is identical for both compounds (2.2(5) × 10^14^ s^–1^). Consequently, the difference between the two lines arises solely from the difference in activation energy (0.49 eV vs 0.41 eV), consistent with the expectation that *N*_c_, rather than ν_max_, is the primary factor compensating for the conductivity difference between the two systems.

In addition to differences in the effective charge-carrier concentration *N*_c_, changes in the microscopic migration mechanism may also contribute to the enhanced Li-ion mobility observed for Li_6.5_La_3_Zr_1.5_Ta_0.5_O_12_. The higher Li content may lead to shorter average Li–Li distances (see above)^30^ and a more densely populated diffusion network. Under such conditions, correlated or even concerted Li-ion motion^76–78^ may become increasingly important, potentially reducing the effective migration barrier and thereby contributing to the higher hopping rates observed in this material. Such a scenario has recently been proposed on the basis of neutron crystal-structure data revealing split occupancy of both the 96*h* and 24*d* voids.^30^ The similarity of the macroscopic conductivities nevertheless suggests that differences in the effective charge-carrier concentration *N*_c_ remain the dominant factor governing the overall conductivity.

In this context, the Li^+^ vacancy concentration, adjusted by the Li content through the chosen Zr:Ta ratio, may play a crucial role in determining *N*_c_. While *N*_c_ itself seems to show no (Zr:Ta, 1:1)^48^ or only a weak temperature dependence (see above), the larger defect or vacancy concentration in Li_6_La_3_ZrTaO_12_ leads to an increased *N*_c_, ultimately resulting in a conductivity highly comparable to that observed for the system with a Zr:Ta ratio of 1.5:0.5.

## Conclusion

In this study, Li-ion dynamics in single-crystalline Li_6.5_La_3_Zr_1.5_Ta_0.5_O_12_ were investigated over a wide frequency window spanning the kHz to GHz range by combining ^7^Li NMR relaxation with conductivity spectroscopy and electric modulus measurements. The joint analysis provides a coherent and frequency-resolved picture of Li^+^ motion, allowing us to bridge local jump processes and macroscopic charge transport under grain-boundary-free conditions. Long-range ionic transport is characterized by activation energies of about 0.41(1) to 0.44(1) eV, as consistently derived from dc conductivity, electrical modulus analysis, and spin-lock NMR relaxation rates. The macroscopic diffusion coefficients reported in the literature by field gradient NMR techniques^30,47,72^ are in excellent agreement with this analysis of ionic transport. In contrast, localized Li^+^ dynamics occur with substantially lower barriers of approximately 0.20 to ca. 0.25 eV, reflecting short-range hopping processes within the Li substructure.

The combined NMR and electrical data thus reveal a heterogeneous energy landscape in which low-barrier local motions coexist with higher-barrier processes governing extended diffusion. Comparison with single-crystalline Li_6_La_3_ZrTaO_12_, see Stanje et al.,^48^ further shows that Li^+^ mobility in Li_6.5_La_3_Zr_1.5_Ta_0.5_O_12_ is higher by roughly 1 order of magnitude. Nevertheless, the resulting ionic conductivities of the two systems remain similar, which can be explained by differences in the effective number density of mobile charge carriers *N*_c_. A higher vacancy concentration in Li_6_La_3_ZrTaO_12_ likely increases *N*_c_, compensating for its lower intrinsic Li^+^ mobility. Whether the Li^+^ occupation disorder^30^ reported for Li_6.5_La_3_Zr_1.5_Ta_0.5_O_12_ contributes to this effect, and whether it promotes concerted Li-ion migration, remains to be clarified by future experimental and computational studies.

Our results show that practical ionic conductivity in garnet electrolytes is governed by both Li^+^ mobility and, critically, the effective carrier concentration; identifying and tuning the latter provides a decisive lever for optimization. Our single-crystal, cross-validated approach links local jump processes to long-range transport across complementary frequency windows, offering actionable guidance for compositional design of oxide solid electrolytes and establishing robust benchmarks for interpreting transport in polycrystalline systems. Overall, combining NMR with electrical spectroscopy enables a comprehensive, internally consistent characterization of ion dynamics in garnet single crystals that can be directly transferred to more complex materials.

## Data Availability

The data that support the findings of this study are presented in the paper and are available from the corresponding author upon reasonable request.
